# Body Posture Asymmetry Differences between Children with Mild Scoliosis and Children with Unilateral Cerebral Palsy

**DOI:** 10.1155/2013/462094

**Published:** 2013-10-09

**Authors:** Małgorzata Domagalska-Szopa, Andrzej Szopa

**Affiliations:** School of Health Sciences, Medical University of Silesia, 12, 40-752 Katowice, Poland

## Abstract

Patients with unilateral cerebral palsy (CP) often have impaired movement coordination, reduced between-limb synchronization, and less weight bearing on the affected side, which can affect the maintenance of an upright weight-bearing position and gait. This study evaluated whether the different postural patterns of children with unilateral CP could be statistically recognized using cluster analysis. Forty-five outpatients with unilateral CP (mean age, 9 years and 5 months) and 51 able-bodied children with mild scoliosis (mean age, 9 years and 2 months) were included. One observer performed moiré topography (MT) examinations using a CQ Electronic System (Poland) device. A weight distribution analysis on the base of support (BOS) between the body sides was performed simultaneously. A force plate dynamographic platform (PDM), ZEBRIS (Germany), with FootPrint software was used for these measurements. Cluster analysis revealed three groups: Cluster 1 (*n* = 71, 73.96%), Cluster 2 (*n* = 8, 8.33%), and Cluster 3 (*n* = 17, 17.71%). Based on the MT parameters (extracted using a data reduction technique), three typical asymmetrical postural patterns were described: (1) the postural pattern of children with mild scoliosis (SCOL), (2) the progravitational postural pattern (PGPP), and (3) the antigravitational pattern. Patterns two and three were identified in children with unilateral CP.

## 1. Introduction

In children with cerebral palsy (CP), atypical body posture patterns (PPs) are observed [1]. They are the effect of functional strategies to compensate for the primary anomalies (i.e., directly attributable to central nervous system damage) and include abnormal muscle tone, abnormal reflex activity, and balance and movement problems. Those attributable to secondary anomalies are compensations the individual uses to circumvent postural problems. Patients with CP have increased cocontraction of agonist and antagonist muscles, a proximal to distal muscle response, and decreased trunk muscle activation [1–4]. Furthermore, patients with unilateral CP tend to have impaired coordination of movement, reduced between-limb synchronization, and less weight bearing on the affected side, which in turn can affect the ability to maintain an upright weight-bearing position as well as gait [5, 6].

A symmetric weight-bearing distribution between the legs during quiet standing provides optimal biomechanical stability, whereas weight shifts prevent the progressive build-up of fatigue in the legs [7]. However, patients with postural deficits, such as CP, might have a different weight distribution between the legs [7, 8]. Postural asymmetry is also commonly associated with scoliosis. Scoliosis is a three-dimensional deformity of the spine characterized by rotations in all three planes of view. There are a variety of different types of scoliosis. The curve may develop secondary to a neuromuscular disorder such as spina bifida or CP, or it may be congenital, due to an underlying abnormality of the formation of the spine. In many cases, no cause of the scoliosis can be determined. This is commonly termed idiopathic scoliosis.

Moiré topography (MT) is an imaging method for the body surface and is highly sensitive in detecting asymmetry [9–11]. Historically, MT was based on the interference of grids projected onto the subject's back [12]; the currently used methods are based on computerized image capturing and digitally calculated parameters. A few studies have reported a high correlation between moiré angle analysis and radiographic analysis of the spinal curvature, in agreement with the results of a previous study by Benoni and Willner [10]. 

Clustering analysis attempts to maximally separate subpopulations by exclusively assigning an instance to only one class. Colloquially, clustering attempts to identify groups of instances, so that the instances within a group are similar to each other while being dissimilar to those instances in all other groups. The most common approach is to use hierarchical cluster analysis and Ward's method. K-means clustering is very different from the above, which are applied when there is no prior knowledge of how many clusters there may be or what they are characterized by. K-means clustering is used when hypotheses concerning the number of clusters in the cases or variables have already been made. K-means cluster analysis is thus a tool of discovery used to reveal associations and structure within data that, although not previously evident, are sensible and useful when discovered [13].

 In our previous study, which presented a descriptive analysis of abnormal postural patterns in children with hemiplegic CP [14], hemiplegic children were observed to have several different strategies for maintaining upright standing posture. They varied from largely relying on their unaffected side for weight support, to standing almost symmetrically, to supporting more weight on their affected leg. Based on the weight bearing between the affected and unaffected body sides and the characteristic relationship between the shoulder and pelvis, two types of asymmetrical postural patterns were described: (1) the progravitational postural pattern (PGPP), with overloading of the affected body side, and (2) the antigravitational postural pattern (AGPP), with underloading of the affected side. 

 There are few studies on asymmetric weight bearing during standing [15, 16] or postural patterns [14] in children with CP, and none have focused on the relationship between them. Therefore, the purposes of this study were to explore the differences between the asymmetry of the body postures of children with mild scoliosis and those with unilateral CP based on MT examinations and to evaluate whether different postural patterns in children with unilateral CP could be statistically recognized with cluster analysis. The expectations were that patients with unilateral CP would have a greater difference in the overall weight bearing between body sides (affected/unaffected) and, specifically, that they would have stronger asymmetries in body posture than children with mild scoliosis. Additionally, the study was designed to verify the hypothesis that children with unilateral CP are not homogeneous in terms of their body weight distribution and body posture patterns. 

## 2. Materials and Methods

The research protocol was approved by the Silesian Medical University Bioethics Committee in Katowice, Poland. The parents/guardians provided signed informed consent prior to the subjects' enrollment in the study. 

### 2.1. Subjects

The study participants were 45 children (17 girls and 28 boys, mean age 9 years and 5 months, range 7 years and 4 months to 12 years and 2 months (SD = 2.11)) with unilateral CP. There were 29 patients with right-sided deficits and 16 patients with left-sided deficits. All participants were outpatients (75.5% Level I and 24.5% Level II by the Gross Motor Function Classification System) at local pediatric rehabilitation centers.

In the reference group, there were 51 able-bodied children with mild scoliosis (27 girls and 24 boys; range of lateral curvature, 11°–25°, mean, 18°; mean age, 9 years and 2 months, range, 7 years and 5 months to 12 years and 3 months (SD = 1,99)). All controls were outpatients at a local Center for Corrective Gymnastics. They were diagnosed by a physician as having idiopathic scoliosis. Twenty-two patients had recently undergone radiographic examination of the spine.

All subjects met the following criteria: (1) older than 7 years of age, (2) able to follow verbal directions, (3) scoliosis (angle of vertebral lateral curvature <20°), and (4) no previous surgical procedures. Additional criteria for subjects with CP were as follows: (1) the diagnosis of spastic hemiplegia, (2) the ability to stand without assistance, (3) not taking any pharmacological agents at the time of the study, and (4) no spasticity management 6 months before the evaluation.

The exclusion criteria were previous orthopedic surgery, severe asymmetrical fixed deformity or scoliosis (angle of vertebral lateral curvature >20°), and dislocation of the hip. Statistical analysis confirmed that the patient demographic characteristics were similar in both groups.

### 2.2. MT Examination

**Figure 1:**
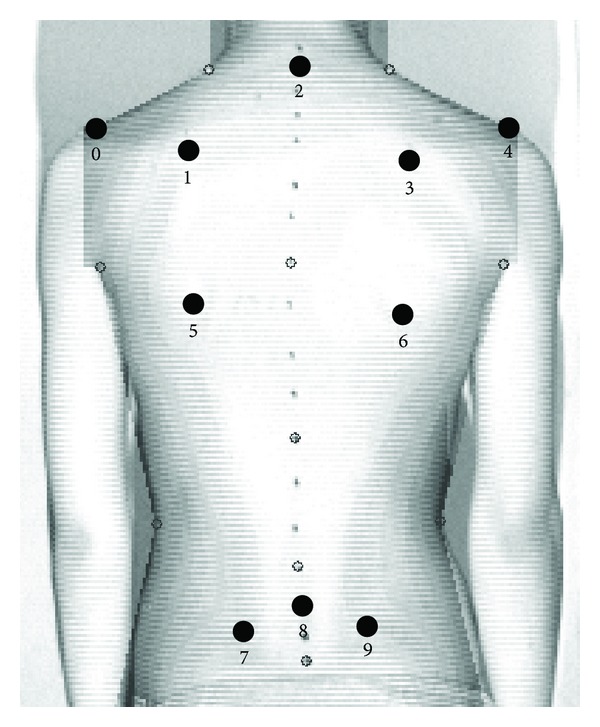
Surface topography parameter settings.

For the MT examination, it was necessary to uncover the entire surface of the back and to mark some anatomical landmarks. These landmarks were the spinous process of C_7_ (2), spinous process of S1 (8), acromial angle of the shoulders (AAOS) (0, 4), superior angle of the scapula (SAOS) (1, 3), inferior angle of the scapula (IAOS) (5, 6), and the posterior superior iliac spine (PSIS) (7, 9), as suggested by the Society on Scoliosis Orthopedic and Rehabilitation Treatment (SOSORT) [17] (Figure 1).

During the examination, the light was turned off, and the child stood quietly with his/her eyes open. The projection angle was 90°, which meant that the camera was placed perpendicularly to the measured surface. The 40 ms images of the back were captured with a CCD camera. An image recording sequence lasted from 5 to 15 seconds. The image most characteristic of the child was chosen for further analysis. 

### 2.3. Data Collection and Analysis

In the literature, many indices are computed in each of the three planes. The following indices were chosen (Figure 1).

Indices measured on the coronal plane are as follows.

1. Spinous process line (SP): the angle of inclination contained between two adjacent lines, a line situated within the sagittal plane and a line of spinous processes from C_7_ through S_1_ (Figure 1: landmarks 2 and 8). The angle value ranged from 0° to 180°.
2. Shoulder line (SHL)*: bilateral SAOS (Figure 1: landmarks 1 and 3).
3. Pelvic line (PL)*: bilateral PSIS (Figure 1: landmarks 7 and 9).
4. Angle of the vertebral lateral curvature (ALC).
5. *D*_max⁡_: the maximum of the horizontal distances measured from the vertical line (VL) from the SP. If the apex of the major curve was on the right side of the VL, the value ranged from −180° to 0°. If the apex of the major curve was on the left of the VL, the value ranged from 0° to 180°.

*The angle of inclination was contained between two adjacent lines: a line situated within the horizontal plane and a line connecting the SAOS (Figure 1: landmarks 1 and 3) and the PSIS (Figure 1: landmarks 7 and 9) lying on the back surface. This line was situated symmetrically on the left and right sides; *α* had a value ranging from −180° to 0° when the right SAOS or PSIS was higher than that of the left or from 0° to 180° when the left SAOS or PSIS was higher than that of the right.

The coronal plane is the major plane for measuring back deformity because it is related to the Cobb angle definition (Figure 1). Because the Cobb angle can only be obtained with X-ray measurements, back surface indices were invented to simulate the Cobb angle.

Indices measured on the transverse plane are as follows.

The angle of rotation was the major index used for the reference to this plane.

(6) Angle of trunk rotation (ATR)**.
(7) Angle of shoulder rotation (SHR)**.
(8) Angle of pelvic rotation (PR)**.

**The angle of surface rotation (*α* angle) was contained between two adjacent lines: a line situated within the frontal plane and a line that connected two points on the back surface and was situated symmetrically on the left and right sides of the corresponding spinous process, the bilateral SAOS, or the PSIS. 

Indices measured on the sagittal plane are as follows.

These indices refer to the location and the magnitude of the maximum kyphosis and lordosis.

(9) The magnitude of the maximum kyphosis (*K*_max⁡_).
(10) The magnitude of the maximum lordosis (*L*_max⁡_).

The MT examination was performed by one observer using a CQ Electronic System (Poland). 

### 2.4. Pedobarographic Measurements (PMs)

An analysis of the weight distribution between the right and left (in Ref) and between affected and unaffected (in SH) body sides was conducted simultaneously with an MT examination. A force plate PDM, ZEBRIS (Germany), with FootPrint software was applied for these types of PMs. Each measurement was recorded three times (3 trials, each lasted for 30 seconds with a 30-second pause between trials), and the most typical measurement of each trial was chosen as the mean weight value for the calculation of weight distribution on the right and left body sides in the reference subjects and on the unaffected/affected body sides in children with hemiplegia for further analysis.

Two experienced physical therapists selected both the moiré photographs and body weight distribution measurement. The image that was most characteristic of the child was chosen for further analysis. When the two experts agreed, the arithmetic mean of their assessments was recorded. When their assessments differed, the senior author (M. Domagalska-Szopa) chose the image that was analyzed. The accuracy of their evaluations was then analyzed.

### 2.5. Data Collection and Analysis

Based on the index of asymmetry (IA) of weight distribution on the unaffected/affected body sides (>40%/60%), the hemiplegic children were divided into four subgroups (four postural patterns) based on the above criteria:

 LL—left side hemiplegic and the tendency to overload the affected body side (*n* = 10, 10.5%);
 RR—right side hemiplegic and the tendency to overload the affected body side (*n* = 13, 13.5%);
 LR—left side hemiplegic and the tendency to overload the unaffected body side (*n* = 6, 6.3%);
 RL—right side hemiplegic and the tendency to overload the unaffected body side (*n* = 16, 16.7%).

Based on the same criteria, the children with scoliosis were divided into two subgroups:

 NL—the tendency to overload the left body side (*n* = 28, 29.2%);
 NR—the tendency to overload the right body side (*n* = 23, 23.8%).

### 2.6. Statistical Analysis

The IA of weight distribution between the right and left body sides was calculated for the controls. The standard deviation was used as a criterion to define the asymmetry of weight distribution on the affected/unaffected body sides in children with SH (IA > 9.83%) to create four SH subgroups (LL, RR, RL, and LR) and two control subgroups (NR and NL).

 Intraclass correlation coefficient (ICC) with 95% confidence interval was used to measure the overall intraobserver and interobserver agreement. Interobserver agreement was calculated separately for each of the MT and PT parameters, based on two examinations performed by the same two researchers in each group (SH and controls) of 10 subjects (40 examinations in total). Interobserver agreement was calculated (for the same subjects) for two of the reviewers. For the analysis, mean ICC values of 0.80 and above reflected excellent reliability, those between 0.70 and 0.79 indicated good reliability, and those below 0.70 reflected poor to moderate reliability. Because of the high dimensionality of the postural analysis data and the correlations between the parameters, a data reduction technique (specifically, factor analysis with six factors extracted) was used as an input for nonhierarchical *k* means clustering. The number of clusters was selected based on a study of the observed overall *R*-squared and the cubic clustering criterion. Three clusters were defined. The means and standard deviations (SDs) of all parameters were calculated for the total group and for each of the three clusters. All data were compared between the subgroups. Analysis of variance (ANOVA) and Tukey's posthoc test were used to detect differences in the MT examination parameters between the three clusters. Only significant differences (*P* < 0.05) between the clusters are described and discussed.

## 3. Results

Using a data reduction technique, five grouping variables were extracted: SP, PL, SHL, ALC, and *D*_max⁡_. According to the cluster analyses, 71 participants (73.96%) were classified into Cluster 1, 8 (8.33%) into Cluster 2, and 17 (17.71%) into Cluster 3. There appeared to be some major differences between the means of the various clusters for each variable, which are shown in Table 1.  Table 2 shows the *F* values and significance levels, which indicate that all differences between the means are significant.

Tukey's posthoc test revealed that five of the MT parameters (excluding ALC) reliably differentiated Cluster 1 and both Clusters 2 and 3 through their cluster means. Three of the MT parameters (PL, SHR, and ALC) demonstrated significant differentiation between Clusters 2 and 3 (Table 2). Cluster 1 was predominantly characterized by the NR and NL subgroups from the reference group (Table 3). Cluster 2 was predominantly characterized by the LL subgroup of the CP-H group (Table 3). Cluster 3 was predominantly characterized by the RL subgroup of the CP-H group (Table 3).

 In this large cohort of children, asymmetrical body posture was recognized in all three clusters. Cluster 1 (*n* = 71, 74%) showed the postural patterns of all children in the SCOL (NR + NL) group (except 1 subject) and a portion of the hemiplegic subjects, primarily those from the RR subgroup: right-sided hemiplegia with a tendency to overload the affected body side (*n* = 13, 18%).

 The average IA of weight distribution between the right/left body sides or the affected/unaffected body sides in children from this cluster indicated almost symmetrical weight bearing (Table 4). Their postural patterns were defined by the largest means of ALC and *D*_max⁡_, which characterized the vertebral lateral curvature, and by the lowest means of the fringe deviations of SP, PL, and SHR. Clusters 2 and 3 were composed only of hemiplegic subjects. Cluster 2 was characterized primarily by hemiplegic patients, who presented a tendency to overload the same affected body side, and these subjects had left-sided hemiplegia (LL, 62.5%). Cluster 3 was composed primarily of hemiplegics with the tendency to overload the unaffected body side (RL, 70.59%). The IA of children from Cluster 3 was extremely high, and each cluster significantly differed from the others (each *P* < 0.0001).

 Significantly higher ALC values (approximately 10°) were noted in subjects in Cluster 2 (Table 2), whereas children in cluster 3 demonstrated significantly higher fringe deviations in the pelvis inclination and shoulder rotation (Table 2). Additionally, S-type curvature was more characteristic of Clusters 1 (76%) and 2 (87%), whereas C-type scoliosis predominated in Cluster 3 (69%).

 Based on the aforementioned relationships, three types of postural patterns in children with body posture asymmetry have been recognized:

1. the asymmetrical postural pattern with almost symmetrical weight bearing (SS),
2. the asymmetrical postural pattern with asymmetrical weight bearing and overloading of the affected body side (+AS),
3. the asymmetrical postural pattern with asymmetrical weight bearing and underloading of the affected body side (−AS).

Every outcome from the MT and PT examinations demonstrated good to very high level of intraobserver agreement for both groups of subjects, with the ICC ranging from 0.72 to 0.96 for CP and from 0.79 to 0.99, in all variables for able-bodied subjects. ICC values indicated very high level of interobserver agreement among researchers, with the ICC ranging from 0.92 to 0.99 for both groups.

## 4. Discussion

Children with asymmetrical body posture have a variety of postural patterns. Cluster analysis was used to recognize different postural patterns and to search for underlying pathological mechanisms that could explain the large intersubject variability in the body postures of children with asymmetry of body posture. Three asymmetrical postural patterns were described based on the weight bearing between body sides and MT parameters, which were extracted using a data reduction technique; these included one pattern with almost symmetrical weight bearing and two different patterns with asymmetrical weight bearing. The asymmetrical postural pattern with almost symmetrical weight bearing was characteristic of all children with moderate scoliosis and for hemiplegic subjects with right-sided hemiplegia and a tendency to overload the affected body side (RR). The cluster analyses also identified two asymmetrical postural patterns with asymmetrical weight bearing in hemiplegic children; one was overloading of the affected body side (+AS), and the other was underloading of this side (−AS).

 Clear differences in the MT parameters were characterized by the spinal deformities and the fringe deviations in pelvic obliquity and the shoulder girdle rotation, which were observed between these three postural patterns. Interestingly, greater spinal deformity was more commonly observed in children with almost symmetrical weight bearing (SS), not in the groups with asymmetrical patterns of weight bearing. Conversely, the pelvic obliquity and shoulder girdle rotation were the most important pathogenic factors in hemiplegic children with asymmetrical weight bearing (AS). Interestingly, hemiplegic subjects with a tendency to overload the affected body side (+AS) were commonly found in the group of children with scoliosis. Most likely, the observed reclustering was due to the higher values of the angle of curvature for scoliosis and the symmetrical weight bearing between the affected and unaffected body sides, which were more typical for children with scoliosis. The postural pattern did not appear to be determined by the diagnosis but primarily by the symmetry/asymmetry distribution of body mass between body sides and the value of scoliosis as well as the spatial relationship between the shoulder rotation and pelvic obliquity. In all subjects exhibiting almost symmetrical weight bearing, scoliosis represented the most common pattern of deformity, whereas children with asymmetrical weight bearing presented with laterality and pelvis obliquity (up) and a large externally rotated shoulder girdle on the affected side. Therefore, the differences between the asymmetrical postural patterns of children with moderate scoliosis and children with hemiplegia were not as clear as expected. 

 Despite the fact that a group of children with spastic hemiplegia appears to be relatively homogeneous, the present study has shown that their postural patterns differ. Based on the MT and the PMs of the body mass distribution between the affected and unaffected body sides, two different postural patterns were recognized in children with unilateral CP, one with overloading of the affected body side (+AS) and the second with underloading of the affected body side (−AS). This finding suggests that the distribution of body mass between the affected and unaffected body sides determined the characteristic compensatory action, which was the spatial relationship between the shoulder rotation and pelvic obliquity and the type and value of scoliosis. The obtained results confirmed the hypothesis that children with unilateral CP are not homogeneous in terms of their body weight distribution and body posture patterns. However, the diversities of the PGPPs and AGPPs described in our previous study were not completely confirmed. This finding should be confirmed in other series of statistical analyses before hypotheses can be formulated regarding this difference.

To our knowledge, this study is the first to examine body posture using MT as an objective evaluation of body posture in children with CP. The data demonstrated a very high intertrial reliability for every variable calculated from the MT examination in both able-bodied children and children diagnosed with CP. Previously, Chowanska and coauthors reported good intraobserver repeatability using CQ surface topography to examine children with scoliosis [12]. In our country, a portable raster stereography device is available (CQ Electronic System) and was used in this study. CQ has been reported to precisely measure the angle of scoliosis as the ALC, but this value is not the same as the Cobb angle. The ALC has a value ranging from 180° to 90°, and the angle of scoliosis is larger when the ALC is close to 90°. Therefore, it is difficult to compare our results with those from other studies that used the Cobb angle. Additionally, plus (+) and minus (−) are often used to characterize the direction of inclination or rotation (see SHL, PL, *D*_max⁡_, ATR, SHR, and PR), not the real value of the deformity indices. Therefore, the mean values did not show any real value of disturbance because the positive (+) and negative (−) values were neutralized within the same parameters. However, the absolute value of these parameters did not inform us of the nature of the deformity (e.g., up or down, internal or external, right or left). Dividing the study participants into six subgroups based on the postural patterns was an important point of our analyses. This breakdown of the material strongly modified the results of the analyses. In this way, two different postural patterns were isolated in children with unilateral CP for the first time.

An asymmetric alignment while standing is often characteristic of children with a unilateral neurological lesion, such as hemiplegia [18]. Despite many studies reporting a postural problem in children with CP, such as postural dysfunction in adapting to the adjustment [1, 19], problems with postural control during standing, walking, and running [6, 20], and anticipatory and compensatory postural adjustments in sitting and standing [21], there are no data examining the body postures of children with CP in the literature. Multiple studies have examined the steady-state balance control and the postural alignment with all characteristics of body sway while standing quietly [4, 22], but only a few studies have examined the body weight distribution between the affected and unaffected sides. An experimental study has indicated that children with unilateral CP tend to displace their weight toward the uninvolved side [6, 18]. However, our current studies have shown that children with unilateral CP are not homogeneous in terms of their body weight distribution and that these observations only apply to certain subgroups of children with AS−. Children expressing AS+ tend to overload their affected sides, and they retain an asymmetrical crouched posture. Asymmetrical weight bearing may be an effect of the different type of brain damage observed in children with unilateral CP, which can lead to the development of two different compensatory postural mechanisms. The asymmetry of body posture is primarily determined by the compensatory mechanisms for deficits in postural control, which are expressed in a particular postural pattern [23].

It is well known that it is not possible to achieve thoroughly correct postural patterns when treating children with CP. The entire rehabilitation process for these children is based on the steering of compensation and alleviating the brain lesion symptoms. Certain consequences of compensatory postural patterns will develop and exceed their natural abilities of acting against gravity; these consequences must be considered in every case [24]. The present study has recognized and defined the mature compensatory postural patterns in children with unilateral CP. This awareness may be essential in the decision making process regarding the management, facilitation, modification, or elimination of each compensatory sign.

## 5. Conclusions

The present study recognized and defined differences between the asymmetry of the body postures of children with mild scoliosis and children with unilateral CP. Additionally, this study demonstrated that despite apparent similarities in children with unilateral CP, their postural patterns differed.

This awareness may be essential in the decision making process regarding the management, facilitation, modification, or elimination of each compensatory sign. The results of this study were promising, and, therefore, these findings should be confirmed in another series of statistical analyses that will precisely define postural patterns in children with unilateral CP and demonstrate the basic differences between them. 

## Figures and Tables

**Table 1:** Parameter descriptions.

MT parameter	Cluster	Mean	*N*	Std deviation	Minimum	Maximum
SP (°)	1	1.5	71	4.0	−4.2	13.4
	2	−3.4	8	5.3	−11.9	3.6
	3	−5.4	17	5.0	−11.3	3.5
	Total	−0.1	96	5.1	−11.9	13.4

PL (°)	1	−1.7	71	5.1	−13.9	10.6
	2	7.6	8	3.9	2.1	13.1
	3	10.2	17	3.3	2.4	14.3
	Total	1.2	96	6.8	−13.9	14.3

SHR (°)	1	1.1	71	9.1	−20.9	18.0
	2	1.3	8	11.8	−16.6	15.9
	3	−11.6	17	10.9	−31.0	2.2
	Total	−1.1	96	10.8	−31.0	18.0

*D*_max⁡_ (mm)	1	−9.5	71	5.9	−17.6	11.2
	2	8.9	8	8.1	−1.5	18.5
	3	7.9	17	4.3	2.8	15.1
	Total	−1.9	96	8.4	−17.6	18.5

ALC (°)	1N	162.9	71	4.4	168.4	156.0
	2PP	166.4	8	3.2	180.0	170.5
	3AP	176.2	17	3.7	180.0	164.5
	Total	176.6	96	5.5	180.0	156.0

MT: moiré topography; SP: spinous process line; PL: pelvic line; SHR: angle of shoulder rotation; *D*_max⁡_: the maximum of the horizontal distances measured from the vertical line to the spinous process line; ALC: angle of the vertebral lateral curvature.

**Table 2:** Results of analysis of variance (ANOVA). Differences between the means of various clusters for MT parameters.

MT parameter	Groups	Sum of squares	df	Mean square	*F*	*P*
SP	Between	743.90	2	18.61	19.99	0.00000
	Within	371.95	93			
	Total	1730.62	95			

PL	Between	2318.34	2	22.80	50.84	0.00000
	Within	1159.17	93			
	Total	2120.50	95			

SHR	Between	2270.67	2	93.64	12.12	0.00002
	Within	1135.33	93			
	Total	8708.94	95			

*D*_max⁡_	Between	3500.70	2	34.56	50.64	0.00000
	Within	1750.30	93			
	Total	3214.34	95			

ALC	Between	1684.60	2	13.34	63.15	0.00000
	Within	842.30	93			
	Total	1240.44	95			

MT: moiré topography; SP: spinous process line; PL: pelvic line; SHR: angle of shoulder rotation; *D*_max⁡_: the maximum of the horizontal distances measured from the vertical line to the spinous process line; ALC: angle of the vertebral lateral curvature.

**Table 3:** Nonhierarchical *k* means clustering.

Subgroup		Cluster 1 NN	Cluster 2 PP	Cluster 3 AP	Total
NR	(*N*)	22	1	0	23
	(%)	30.99	12.50	0.00	23.96
NL	(*N*)	28	0	0	28
	(%)	39.44	0.00	0.00	29.17
RR	(*N*)	13	0	0	13
	(%)	18.31	0.00	0.00	13.54
RL	(*N*)	2	2	12	16
	(%)	2.82	25.00	70.59	16.67
LR	(*N*)	6	0	0	6
	(%)	8.45	0.00	0.00	6.25
LL	(*N*)	0	5	5	10
	(%)	0.00	62.50	29.41	10.42
Total	(*N*)	71	8	17	96
	(%)	73.96	8.33	17.71	100.00

Two subgroups of children with scoliosis. NL: the tendency to overload the left body side; NR: the tendency to overload the right body side and four subgroups of children with CP; RR: right side hemiplegic and the tendency to overload the affected body side; RL: right side hemiplegic and the tendency to overload the unaffected body side; LL: left side hemiplegic and the tendency to overload the affected body side; LR: left side hemiplegic and the tendency to overload the unaffected body side.

**Table 4:** Summary of the index of asymmetry of weight distribution between right/left body sides in control subjects and the affected/unaffected body sides in children with hemiplegia in particular clusters.

Cluster	Index of asymmetry				
	Mean (%)	*N*	SD (%)	Minimum (%)	Maximum (%)
Cluster 1	−1.45	51	9.83	−18.00	22.00
Cluster 2	6.96	23	28.48	−38.00	48.00
Cluster 3	−12.25	22	26.75	−46.00	46.00
Total	−1.91	96	20.99	−46.00	48.00
